# Supplementary material for: Microbial Residents of the Atlantis Massif’s Shallow Serpentinite Subsurface
Source: Appl Environ Microbiol. 2020 May 19;86(11):e00356-20. doi: 10.1128/AEM.00356-20 (PMC7237769; doi:10.1128/AEM.00356-20)
Supplement: Supplemental file 1 [file AEM.00356-20-s0008.pdf]

Supplementary Materials

Supplement to:

Microbial residents of the Atlantis Massif's shallow serpentinite subsurface

Shahrzad Motamedi, Beth N. Orcutt, Gretchen L. Früh-Green, Katrina I. Twing, Heather L. Pendleton, and William J. Brazelton

Content:

1. Supplementary methods description
2. DNA extraction and purification from low-biomass rocks
3. DNA extraction from Sterivex filters
4. 16S data processing with DADA2

1. Supplementary methods description

Sampling Location

Below is a brief summary of the sampling process; further details are available elsewhere (1, 2). During IODP Expedition 357 to the Atlantis Massif (Mid-Atlantic Ridge 30°N), rock cores were collected from seventeen drilled holes at nine sites (**Figure 1**): two sites on the eastern end of the southern wall (Sites M0068 and M0075), three sites in the central section of the southern wall north of the Lost City hydrothermal field (Sites M0069, M0072, and M0076), and two sites on the western end (Sites M0071 and M0073. Onshore investigations of the split cores indicated that the central sites preserved in-situ basement

sequences, whereas the cores from the eastern and western sites consisted of talus blocks eroded from the top of the massif during uplift but showing a similar alteration history as the in-situ sequences (2). Rock and water samples were collected for a wide range of geological, physical, chemical, and biological measurements by the scientific party, as described in (3). In this report, we describe new environmental DNA sequencing results from the rock cores and water collected during the expedition.

Description of Rock Samples

Two seabed drill systems were utilized during this expedition: the British Geological Survey (BGS; Edinburgh, United Kingdom) RockDrill2 (RD2) and the Center for Marine Environmental Sciences (MARUM; Bremen, Germany) Meeresboden-Bohrgerät 70 (MeBo70). Both drills are remotely operated systems that are lowered onto the seabed, with power and control maintained from the surface via an umbilical cable. Additional details on the drill systems were provided in previous reports (2, 3). More than 57 m of cores were recovered by the drill systems, with borehole penetration ranging from 1.30 to 16.44 m below seafloor and core recoveries as high as 74.76% of total penetration (2). For microbiological investigations, a 2–20 cm section of whole round core from one end was selected for pooled analyses to be completed on shore. The piece was typically divided into two portions, one of which was flame-sterilized with a handheld butane torch inside a flame-sterilized steel box to remove surface contamination. The serpentinite rock samples included in this study that were flame-sterilized are 0AMRd010, 024, 031, 033, 048, 049, 058, and 072 (**Data set S1**). All rock core samples for microbiological analyses

were stored at -80°C on the ship and then shipped on dry ice directly to the JAMSTEC Kochi Core Center in Japan for processing.

The rock core samples varied greatly in mineralogy, texture, and structure (3). Rock types included serpentinized harzburgites and dunites, intruded by gabbros and dolerite dikes, as well as minor basaltic rocks. In addition, talc-rich rocks (talc-amphibole schists) were typically recovered at contacts between the serpentinites and mafic rocks and are considered to be the product of Si mobility during alteration (3, 4). Some samples were very hard, with very few veins or fissures, and remained as intact cores until subsampling, while others were already rubbly and soft immediately after recovery from the seafloor. The exteriors of intact pieces were shaved off with a steam-sterilized band saw on a frozen stage in a microbiologically clean room at the Kochi Core Center, leaving only the pristine interior sections for downstream microbiological analyses. Shaving the exteriors of fractured and rubbly samples was not practical, therefore these samples were washed three times with ultra-pure, sterilized water before additional processing.

Of the 89 rock core subsamples dedicated for microbiology studies, 35 were processed for this study. These 35 rock samples included 18 characterized as serpentinites with varying talc overprinting, and the DNA sequencing results for 15 of these serpentinite samples exceeded our data quality thresholds (see below). Each rock sample was assigned a standardized IODP sample ID upon recovery that includes the expedition number, site, hole, core number, core type, section number, piece number (for hard rock), and interval in centimeters measured from the top of the section. For example, “357-69A-

3R-2, 35–40 cm" is a sample removed from the interval 35–40 cm below the top of Section 2, Core 3R, from Hole M0069A during Expedition 357. During DNA extraction and sequencing in the lab in Utah, rock core subsamples were assigned new IDs: e.g. "0AMRd005" where AMR stands for Atlantis Massif Rock. Each rock sample is labeled with both IDs in **Data set S1**.

DNA Extraction from Rocks

At the Kochi Core Center, after sawing or washing the exteriors of the frozen rock samples, each rock core sample was homogenized into fine grains with a sterile mortar and pestle, as described elsewhere (1). The resulting rock powders were subsectioned into sterile plastic tubes under a HEPA-filtered processing workstation and then refrozen, then shipped on dry ice to the shorebased laboratory in Utah where they were processed in a dedicated room supplied with filtered air and low levels of dust particles. Moreover, two molecular workstations with UV lamps (UVP Model UV3 HEPA PCR Cabinet, Analytik, Jena, Germany; and AirClean Model 600 PCR Workstation, AirClean Systems, Creedmore, North Carolina, USA) were used for aliquoting the rocks into microcentrifuge tubes and for sensitive stages during the DNA extraction, washing, and purification.

DNA was extracted with a custom protocol that involving neither commercial extraction kits nor phenol. All reagents were prepared in our laboratory with molecular-grade powders and ultra-pure water. Development of our protocol benefitted from previous reports on the optimization of DNA extraction and purification methods (5–11). First, 0.5 g of rock powder was placed in 2 mL sterile tubes and mixed with 1000 μL of DNA

Extraction Buffer (0.03M Tris-HCl, 0.01M EDTA, 0.02M EGTA, 0.1M KH_2PO_4, 0.8M guanidine HCl, 0.5% Triton-X 100, pH 10), 150 µL of 20% sodium pyrophosphate solution, and 150 µL of 50 mM dATP (Jena Bioscience, Jena, Germany). The DNA extraction buffer was filtered sterilized, boiled in a microwave for two minutes, and exposed to UV light for 30 minutes in a 50 mL glass bottle before adding to the rock powder. The tubes were shaken and vortexed briefly before an overnight incubation at 4°C, which is thought to allow time for the chelation of salts as well as for the prevention of DNA adsorption to phyllosilicates and other minerals by saturating binding sites with dATP and pyrophosphate. The DNA extraction buffer was verified to contain below-detection levels of DNA with the Qubit 2.0 fluorometer with dsDNA (High-Sensitivity) assay kit (Thermo Fisher Scientific, Waltham, Massachusetts, USA).

The following day, tubes were frozen at -80°C and then thawed at 54°C, 600 rpm for an hour in a shaking Thermomixer. This freeze-thaw step was followed by increasing the heat to 75°C and the speed to 1500 rpm for 30 min. Tubes were beaten in a MiniBeadBeater-16 Model 607 (Biospec, Bartlesville, Oklahoma, USA) (40 s at 3450 oscillations/min). No beads were added for the beating step in order to avoid unnecessary contamination and because our initial tests indicated that the DNA yield did not improve by adding commercial beads to a preparation of crushed rocks. No enzymes were added to extractions in order to avoid additional contamination. Tubes were centrifuged (3 min, 6100 rcf), and the supernatant was transferred to clean tubes. For each rock sample, 3-5 replicate extractions were performed. At this point, DNA in the lysates was quantified with the Qubit 2.0 fluorometer with HS dsDNA assay kit, and the total DNA yield per gram

of rock was calculated by summing the quantifications of all replicate extractions for each sample and dividing by the total grams of rock sample included in the extractions (**Table 1**). The DNA detection limit per gram of rock sample was determined for each sample by calculating the total maximum DNA yield that could have been achieved by the extraction while remaining below the detection limit of the Qubit 2.0 fluorometer (0.05 ng per μL).

The accuracy of DNA quantification was tested by spiking 1000 ng of *E. coli* DNA into four crushed rock samples from the Coast Range Ophiolite Microbiological Observatory (CROMO) in California (12). These samples were subjected to the same DNA extraction and quantification procedure as described above, resulting in 1419-1859 ng of DNA in their crude lysates. These results are roughly consistent with the expectation of obtaining 1000 ng of *E. coli* DNA plus ~800 ng of environmental DNA from these particular rock samples (based on prior experiences with these samples).

Washing and Purification of DNA from Rocks

In order to minimize the loss of DNA, we did not perform phenol extractions or ethanol precipitations. Instead, crude lysates were washed with Vivacon2 filtration units (100,000 MWCO) (VIVACON 2-PCR Grade (ETO), Sartorius, Göttingen, Germany) by centrifugation for 35 min at 2,500 rcf and then washed with 65°C sterile TE (Tris-10mM, EDTA-1mM) and then 65°C ultra-pure water. The VivaSpin membranes are expected to retain DNA molecules larger than 600 bp, which we verified with gel electrophoresis of test samples. DNA in the washed preparations was again quantified with the Qubit 2.0 fluorometer with HS dsDNA.

SCODA (synchronous coefficient of drag alteration) technology implemented with the Aurora purification system (Boreal Genomics, Vancouver, British Columbia, Canada) was used for purification and concentration of DNA from these extremely low-biomass samples. The procedure was conducted with a modification of the manufacturer's standard protocol. The Aurora cartridge and dams were soaked in 10% household bleach (5% sodium hypochlorite) for thirty minutes and thoroughly washed with Milli-Q water (MilliporeSigma, Burlington, Massachusetts, USA). Both cartridge and dams were placed under a UV lamp for an hour. The dams were inserted into their proper locations on the cartridge, and the molten agarose was poured into the designed gap between the dams. The cartridge with molten agarose was exposed to UV light for 15-20 min. The cartridge buffer chambers, and extraction well were filled with 0.25x TBE Buffer. The extraction well was sealed with PCR tape. A pre-run protocol was conducted prior to every run to remove any possible contaminating DNA in the Aurora cartridge.

The sample, after achieving the required level of conductivity (≤100 μS/cm) after washing with the Vivacon2 filter, was loaded into the sample chamber in the cartridge and was placed onto the cold plate in the Aurora instrument. The "106-0001-CA-D_AURORA_DNA_CLEAN- UP_PROTOCOL" was selected in the Aurora software, which is expected to recover DNA molecules 0.3-50 kb in length. The run completes in 4 hours, and the purified DNA was extracted from the concentration well. The output volume was 40-70 μl for each run, and each replicate extraction was processed on a separate Aurora run. Then, the purified outputs of all replicate extractions were pooled for a final

run to concentrate and further purify a final DNA prep for that rock sample. The final result of this protocol is that tiny quantities of DNA from multiple aliquots of large volumes of crushed rock were concentrated and purified in a single, final preparation. The DNA in the final, purified preparation was again quantified with the Qubit fluorometric method.

Extraction of DNA from Seawater

Prior to the deployment of the drill and beginning of drilling operations at each site, a cast of the ship's conductivity, temperature, and depth (CTD) Niskin bottle rosette was undertaken to capture a water column profile, focusing on the bottom water (2). The ship's wireline CTD rosette with six 10 L Niskin bottles was used to collect water from as close to the seafloor as possible (generally 2–3 m above seafloor). Three of the 10 L bottles were triggered near the seafloor at each site. An additional three bottles were triggered at several shallower depths in the water column at each site. Additionally, a 4 L bucket was used to collect surface water near the ship. A total of 76 seawater samples were collected for DNA analysis. These samples include 20 that were collected from "shallow water" (depth of 200-600 meters below the sea level (mbsl)), 37 from "deep water" (depth of 700-1600 mbsf), 19 collected from "surface water" (with a bucket from the ship or with the CTD Niskin bottles from a few meters below the surface). The details related to sample names, site and hole numbers, as well as cast numbers can be found in **Data set S2**. The shallow and deep water samples were collected from CTD Niskin bottles into pre-cleaned 4 L cubic containers and stored at 4°C until further processing. All water samples (surface, shallow, and deep) were filtered through 0.22 μm mesh Sterivex filter cartridges (Millipore, Billerica, Massachusetts, USA) using a peristaltic pump (Model

07518-60, Cole-Parmer, Vernon Hills, Illinois, USA); 1–4 L of fluid was filtered in duplicate per sample. Sterivex filter cartridges were frozen at –80°C and shipped on dry ice to shore-based laboratory in Utah, where they were extracted following a previously described method for DNA extraction and purification (13).

Extraction of DNA from Air

Air from the general lab area and the clean room in the shore-based laboratory in Utah was filtered through 0.1 μm Puradisc 25 mm PTFE syringe filters (GE Healthcare Whatman, Pittsburgh, Pennsylvania, USA) by a dual head Air Cadet Model 420-2901-00FK (Thermo Fisher Scientific, Waltham, Massachusetts, USA). The air was vacuumed through the filter for 9 h each time. The air was filtered in triplicate, and replicates were combined during the DNA extraction. DNA extraction and purification were performed on the three air samples following the same protocols and using the same reagents as for the rock samples. Therefore, the air samples also serve as extraction blanks for comparison with the rock samples.

DNA Sequencing and Sequence Analyses

Bacterial 16S rRNA gene amplicon sequencing was conducted by the Michigan State University genomics core facility on all of the samples (rock, water, and lab air). All samples were sequenced twice (i.e. sequencing replicates). The V4 region of the bacterial 16S rRNA gene was amplified with dual-indexed Illumina fusion primers (515F/806R) as described elsewhere (14). Amplicon concentrations were normalized and pooled using

an Invitrogen SequalPrep DNA Normalization Plate. After library quality control (QC) and quantitation, the pool was loaded on an Illumina MiSeq v2 flow cell and sequenced using a standard 500 cycle reagent kit. Base calling was performed by Illumina Real Time Analysis (RTA) software v1.18.54. Output of RTA was demultiplexed and converted to fastq files using Illumina Bcl2fastq v1.8.4.

16S rRNA gene amplicon sequences were processed with cutadapt v. 1.15 (15) and DADA2 v. 1.10.1 (16) according to a protocol available at https://github.com/Brazelton-Lab/Atlantis-Massif-2015. This protocol includes quality trimming and filtering of reads, removal of chimeras, and inference of amplicon sequence variants (ASVs). Rock and air samples containing fewer than 500 total counts after quality filtering, and water samples containing fewer than 2000 total counts after quality filtering, were excluded from further analyses. Taxonomic classification of all ASVs was performed with DADA2 using the SILVA reference alignment (SSURefv132) and taxonomy outline (17). Rock samples included 287 ASVs (14% of the total ASVs in rocks) that were classified as Archaea. Because the focus of this study was to identify a list of likely rock-inhabiting microbes and not statistical comparisons of bacterial communities, we chose to include the archaeal ASVs in subsequent analyses. Raw counts were converted to proportions to normalize for variations in sequencing depth among samples. The proportional abundances of all 17,081 unique ASVs among all rock, water, and air samples were used to calculate the Morisita-Horn community dissimilarity between each pair of samples. Similar results were obtained with other metrics of dissimilarity (e.g. Bray-Curtis). The non-metric multi-dimensional scaling (NMDS) plot was generated from a table of ASV proportional

abundances across all sample categories (rocks, water, lab air) using the distance, ordinate, and plot_ordination commands in the R package phyloseq v.1.26.1 (18). The major sources of contamination and the level of contamination in each sample were estimated with SourceTracker2, version 2.0.1 (19). Serpentinite samples with >15% of sequences attributed to lab air were excluded from further analyses (0AMRd014A, 031C, 033A, 033C, 034A, 036A, 045B, 067C, 071A, and 073A). In addition, only one of each pair of sequencing replicates was included in further analyses in order to avoid pseudo-replication in later statistical tests and because this only affected three samples (0AMRd030, 057, and 072) at this stage. In each case, the replicate with lesser air contamination was included.

Differential abundance was tested with the R package edgeR v. 3.24.3 (20) as recommended elsewhere (21). We used edgeR to contrast the total read counts of ASVs in serpentinite rock samples compared to three groups of water samples (surface, shallow, deep). ASVs that were absent in all serpentinites and ASVs with low variance (<1e-6) were excluded from the comparisons. The output of the three edgeR tests (serpentinite samples compared to each of the three groups of water samples) was three lists of ASVs with significant differential abundances (false discovery rate (FDR) < 0.05) in serpentinite samples or water samples. The final list of ASVs was created by deleting ASVs with greater abundances in any of the categories of water samples (as determined by the edgeR tests) from the original list of ASVs from which all air ASVs had already been removed. Finally, rare ASVs (those that did not have >=100 counts in a single sample) were excluded from the final results, merely as a conservative abundance filter

for reporting a final list of ASVs expected to be present in serpentinite rocks. No comparisons of diversity were attempted after removing rare ASVs.

References

1. Orcutt BN, Bergenthal M, Freudenthal T, Smith D, Lilley MD, Schnieders L, Green S, Früh-Green GL. 2017. Contamination tracer testing with seabed drills: IODP Expedition 357. Sci Drill 23:39–46.

2. Früh-Green GL, Orcutt BN, Green SL, Cotterill C, Morgan S, Akizawa N, Bayrakci G, Behrmann J-H, Boschi C, Brazleton WJ. 2017. Expedition 357 methods. Atlantis Massif: Serpentinisation and life. Proc Int Ocean Discov Program 357.

3. Früh-Green GL, Orcutt BN, Rouméjon S, Lilley MD, Morono Y, Cotterill C, Green S, Escartin J, John BE, McCaig AM, Cannat M, Ménez B, Schwarzenbach EM, Williams MJ, Morgan S, Lang SQ, Schrenk MO, Brazelton WJ, Akizawa N, Boschi C, Dunkel KG, Quéméneur M, Whattam SA, Mayhew L, Harris M, Bayrakci G, Behrmann J-H, Herrero-Bervera E, Hesse K, Liu H-Q, Ratnayake AS, Twing K, Weis D, Zhao R, Bilenker L. 2018. Magmatism, serpentinization and life: Insights through drilling the Atlantis Massif (IODP Expedition 357). Lithos 323:137–155.

4. Früh-Green GL, Orcutt BN, Green SL, Cotterill C, Morgan S, Akizawa N, Bayrakci G, Behrmann JH, Boschi C, Brazleton WJ. 2017. Expedition 357 summary. Proc Int Ocean Discov Program 357.

5. Direito SO, Marees A, Röling WF. 2012. Sensitive life detection strategies for low-biomass environments: optimizing extraction of nucleic acids adsorbing to terrestrial and Mars analogue minerals. FEMS Microbiol Ecol 81:111–123.

6. Saeki K, Sakai M. 2009. The influence of soil organic matter on DNA adsorptions on andosols. Microbes Environ 0904270086–0904270086.

7. Lever MA, Torti A, Eickenbusch P, Michaud AB, Šantl-Temkiv T, Jørgensen BB. 2015. A modular method for the extraction of DNA and RNA, and the separation of DNA pools from diverse environmental sample types. Front Microbiol 6:476.

8. Barton HA, Taylor NM, Lubbers BR, Pemberton AC. 2006. DNA extraction from low-biomass carbonate rock: An improved method with reduced contamination and the low-biomass contaminant database. J Microbiol Methods 66:21–31.

9. Schrenk MO, Kelley DS, Delaney JR, Baross JA. 2003. Incidence and diversity of microorganisms within the walls of an active deep-sea sulfide chimney. Appl Env Microbiol 69:3580–3592.

10. Tanaka T, Sakai R, Kobayashi R, Hatakeyama K, Matsunaga T. 2009. Contributions of phosphate to DNA adsorption/desorption behaviors on aminosilane-modified magnetic nanoparticles. Langmuir 25:2956–2961.

11. Brazelton WJ, Ludwig KA, Sogin ML, Andreishcheva EN, Kelley DS, Shen C-C, Edwards RL, Baross JA. 2010. Archaea and bacteria with surprising microdiversity show shifts in dominance over 1,000-year time scales in hydrothermal chimneys. Proc Natl Acad Sci 107:1612–1617.

12. Crespo-Medina M, Twing KI, Kubo MD, Hoehler TM, Cardace D, McCollom T, Schrenk MO. 2014. Insights into environmental controls on microbial communities in a continental serpentinite aquifer using a microcosm-based approach. Front Microbiol 5:604.

13. Brazelton WJ, Thornton CN, Hyer A, Twing KI, Longino AA, Lang SQ, Lilley MD, Früh-Green GL, Schrenk MO. 2017. Metagenomic identification of active methanogens and methanotrophs in serpentinite springs of the Voltri Massif, Italy. PeerJ 5:e2945.

14. Kozich JJ, Westcott SL, Baxter NT, Highlander SK, Schloss PD. 2013. Development of a dual-index sequencing strategy and curation pipeline for analyzing amplicon sequence data on the MiSeq Illumina sequencing platform. Appl Env Microbiol 79:5112–5120.

15. Martin M. 2011. Cutadapt removes adapter sequences from high-throughput sequencing reads. EMBnet.journal 17:10–12.

16. Callahan BJ, McMurdie PJ, Rosen MJ, Han AW, Johnson AJA, Holmes SP. 2016. DADA2: High-resolution sample inference from Illumina amplicon data. Nat Methods 13:581–583.

17. Pruesse E, Peplies J, Glöckner FO. 2012. SINA: Accurate high-throughput multiple sequence alignment of ribosomal RNA genes. Bioinformatics 28:1823–1829.

18. McMurdie PJ, Holmes S. 2013. phyloseq: An R Package for Reproducible Interactive Analysis and Graphics of Microbiome Census Data. PLOS ONE 8:e61217.

19. Knights D, Kuczynski J, Charlson ES, Zaneveld J, Mozer MC, Collman RG, Bushman FD, Knight R, Kelley ST. 2011. Bayesian community-wide culture-independent microbial source tracking. Nat Methods 8:761–763.

20. Robinson MD, McCarthy DJ, Smyth GK. 2010. edgeR: a Bioconductor package for differential expression analysis of digital gene expression data. Bioinformatics 26:139–140.

21. McMurdie PJ, Holmes S. 2014. Waste Not, Want Not: Why Rarefying Microbiome Data Is Inadmissible. PLOS Comput Biol 10:e1003531.

2. Extraction and purification from low-biomass rocks

Provenance: adapted from the Schrenk *et al.* (2003) method for sulfides, the Brazelton *et al.* (2010) method for carbonates (which was modified from Barton *et al.* (2006)), and informed by Lever *et al*. (2015) (see above sections for references).

The primary author of this protocol is Shahrzad Motamedi, with contributions from August Longino and Mac Pierce. Steps involving the Aurora system are based on the Aurora manual (Boreal Genomics).

Sample preparation:

- Rock hammer
- Flame sterilized pestle and mortar
- Whirlpack sterile sample collection bags
- 2mL sterile tubes
- Single use sterilized plastic spatula

- 50mL Falcon tubes

Lysis:

- Filter-sterilized and boiled DNA Extraction Buffer (DEB)
- Filter-sterilized and boiled 20% sodium pyrophosphate solution
- ThermoMixer at 54ºC and 75ºC, speed of 600 and 1500 rpm
- 2mL sterile tubes
- MiniSpin 5,000g (rcf)

Bead beating (for the samples with higher DNA concentration)

- 2mL sterile tubes
- Bead beater

Washing sample with VivaSpin column (100,000 MW)

- 70ºC filter-sterilized and boiled TE and ultra-pure water
- Eppendorf Centrifuge 2,500 g (rcf)
- 2mL sterile tubes

Measuring conductivity:

- Distilled water
- Kimwipes
- Standard solution
- Moisture solution
- Laquatwin conductivity meter

Measuring DNA concentration:

- Invitrogen Qubit 2.0 fluorometer
- Qubit dsDNA (High-Sensitivity) Assay Kit
- 500 µL thin-walled PCR tubes

Aurora Purification/Optional Pre-Run:

- Agarose powder
- 0.25x TBE Buffer
- 50 and 15 mL Falcon tube
- Ultra-pure water
- Milli-Q water
- Aurora reusable cartridge
- Cartridge dams

- 10% concentration bleach
- Laminar flow hood
- Incubator at 65ºC
- 500 mL Polystyrene cell culture flask
- PCR tape
- Sealing tool
- Forcep
- Cleaning brush

Recipes:

Low Biomass DNA Extraction Buffer (different from the one uses for Sterivex) for 45 mL:

0.03M Tris-HCl (pH 8)	1.35 mL of 1.0 M
0.01M EDTA	0.135 g
0.02M EGTA	0.342 g
0.1M KH_2PO_4 (pH 8)	0.54 g
0.8M guanidine HCl	3.44 g
0.5% Triton-X 100	0.225 mL (225 µL) of 100%

Add ultra-pure water to ~40 mL
Add NaOH to pH 10
Add ultra-pure water to 45 mL
Use 0.2 µm filter and a syringe to remove possible spores

Boil the buffer in microwave for 2.5 minutes with Med-Hi power in a 1000ml glass bottle, tight the lid in order to not losing any liquid during evaporation. Aliquot into sterile microfuge tubes.

20% Sodium pyrophosphate solution: 2 g sodium pyrophosphate decahydrate, 10 mL ultra-pure water

For 10 mL:

Add the provided amount of sodium pyrophosphate decahydrate and water into the 15 mL Falcon tube and vortex it pretty well and place it in the 70°C incubator for 10 min. ~ Vortex it occasionally during this time. After it dissolve completely, filter-sterilize and boil it along with the DEB buffer.

0.25x TBE Buffer: 1.25 mL 10x TBE Buffer, 48.75 mL ultra-pure water for 50 mL:

Add the provided amount of filtered buffer and water into a 1000ml glass bottle and boil it for 3 minutes with Med-Hi power in the microwave, then pour the buffer in a polystyrene cell culture flask and place it under the UV light under the lamina flow hood for couple of hours. You may need to rotate the flask after each 30 minutes in order to let the UV penetrate the buffer pretty well. This is the minimum required amount for a single Aurora run. So, if you plan to do a multiple run or you want to keep more buffer for later, you need to make more. This buffer is used for both making the agarose gel and running the Aurora.

1% Agarose Gel: 0.05 g Agarose powder, 5 mL 0.25x TBE (made in the previous step) Each run uses approximately 4 ml of agarose. Preparing 5 ml is preferential in case extra agarose is needed (e.g. sealing the edge of each well that contact with the gel)

For 5 mL:
Weight 0.05 g of the dry agarose powder in a 15 mL Falcon tube. Add 5 ml of the 0.25x TBE Buffer. Vortex to ensure no agarose is clumped together.
Microwave approximately 9-11 seconds with lid slightly loosened, until mix is about to boil.
Tighten the lid of the Falcon tube, vortex and incubate the molten agarose at 90-95°C for 1 hour. This leads to improved DNA recovery and it is recommended that this step is not omitted.
After 1 hour, transfer the agarose to a water bath at 60-70°C and leave the agarose in the water bath until use.

****Shouldn’t use the molten agarose that is stored for more than 3 days****

<u>Extraction Protocol</u>

Hammering, grinding, preparing samples

1. Remove sample from freezer. Use rock hammer to crush a small sized chunk of rock into chunks as small as possible under the hood. Use whirlpak bags to avoid contamination. Crush 15-20 grams of sample.
2. Use the flame sterile mortar and pestle to crush the sample into a fine powder. Do this for all 15-20 grams.
3. Pour crushed sample into 50 mL Falcon tube, mix well and store at -80 C. This is your working sample.
4. When ready, measure out 0.5 g of sample into 2 mL sterile tubes.

5. Add 1000 µL DEB, 150 µL 20% sodium pyrophosphate solution, and 150 µL dATP to each tube.
6. Shaking them and vortexing them couple of times before put them in the fridge.
7. Store at 4ºC overnight to allow sample to become immersed in the buffer. Occasional vortexing is recommended during this period.

Possible Stopping Point. Store at -20ºC after the initial overnight period at 4ºC

Lysis

1. Remove tubes from fridge, vortex them and invert them couple of times.
2. Put them in -80ºC freezer for 30'.
3. Remove the tubes from the freezer and place them in Thermo Mixer at 54º C with 600 rpm speed for 45'-1 h.
4. Increase the temperature to 75º C and the speed to the maximum (1500 rpm) and let them stay in the thermomixer for 30'.

You can do beating step (40") without bead for samples with higher DNA concentration or for targeting the G+ bacteria. However, it might increase the conductivity of the samples by let more salt free in the supernatant

5. Centrifuge them with MiniSpin for 3' at 6,100 g/rcf.
6. Transfer the supernatant (fluid) to a new sterile 2 mL tubes. (do this really carefully and make sure that your tip doesn't touch the muddy precipitate)

Possible Stopping Point. Store at -20ºC

Washing sample with VivaSpin column (100,000 MW)

1. Before starting: filter sterilize TE with 0.2 µm syringe filter and boil it.
2. Keep the TE and ultra-pure water in incubator at 65ºC.
3. Load the samples into the columns.
4. If the volume of your samples would be less than 2 mL, add 70ºC TE to bring total volume up to 2 mL.
5. Assemble the columns as it is shown in the manual.
6. Spin for 35' at 2,500 g (not rpm).
7. Empty filtrate container and refill the columns with 2 mL 70ºC TE.
8. Spin again for 35' at 2,500 g.
9. Empty the container again and refill the columns with 2 mL 70ºC ultra-pure water.
10. Spin for 30' at 2,500 g.

11. Remove filtrate tube, invert the concentrator body and insert the recovery cap (the smaller one) into the tube.
12. Spin for 2,500 g for 5’.
13. The recovery caps can be sealed for the storage or you can transfer your clean sample into a new sterile microfuge tube.

Tip:

The column can be pre-rinsed with ultra-pure water before loading your sample.

Measuring conductivity:

1. Dilute the sample with ultra-pure water. The total amount of sample should be up to 5 mL for each Aurora run. So, you can add 4 mL or more of water to the sample. (usually after the washing step you will get something ~200 µL)
2. Open the protection lid of the conductivity meter. Pour some drops of the sample (~100 µL) on the sensor.
3. Press the MEAS button to enter the measurement mode.
4. When it lights up, the measurement is completed.
5. To lock the measured value, press the MEAS button again.
6. The sample conductivity needs to be 100 µS/cm for DNA clean-up run with Aurora.≤
7. After measurement, clean the sensor with DI water and dry it by tapping it gently on some Kimwipes.
8. Turn off the power and close the protection cover before storage.

Make sure to store the sensor without any moisture**

Tips:

1. If you are going to use the conductivity meter after a long storage, you need to put some drops of the moistening solution. Let it sit for 10 min, then wash the sensor with DI water. After this treatment you can turn the meter on.
2. If you want to recalibrate the meter, you should pour the 1.41 mS/cm standard solution. Press the CAL button over 2 seconds. When the CAL and smiley face sign light up, the calibration is completed. Clean the sensor with DI water before loading your sample.

Measuring DNA concentration:

1. For preparing the Qubit Working Solution, the Qubit reagent need to be diluted 1:200 in Qubit buffer. 200 µL of Working Solution is required for each sample.
2. Ensure all reagents are at room temperature.
3. Add 198 µL of the Working Solution and 1 µL of your sample to a 500 µL thin-walled PCR tube.
4. Vortex the tube for 2-3 second.
5. Incubate the tube for 2 min at room temperature.
6. Insert the tube in the Invitrogen Qubit 2.0 fluorometer, select “DNA” and “dsDNA high sensitivity” setting.
7. Select “no” when asked whether to read new standards.
8. Press “read next sample”.
9. Select “calculate stock concentration”.
10. Select “2 µL” for stock volume.
11. Do the math for final volume of your sample for the total DNA concentration.

Make sure to store the dye in the dark all the time

Purification with Aurora instrument

1. Soak the cartridge and dams in 10% household bleach (5% sodium hypochlorite) for thirty minutes. Do not soak any items longer than 30 minutes; this can damage the equipment.
2. Wash the cartridge and the dams with Milli-Q water thoroughly.

****Do not use ethanol to clean cartridge; it will cause the cartridge to crack****

3. Place both cartridge and dams under the UV hood for 30 minutes, or until dry.
4. Insert dams into their proper locations (see “Aurora Reusable Cartridge Handling Manual”) and pour molten agarose into the designed gap between the dams as it is shown in the manual.
5. Be sure to check for, and correct, any abnormalities such as **bubbles** in the molten gel.
6. Expose the cartridge with molten agarose to UV for 15-20 minutes until agarose sets.
7. Once gel is ready, gently remove dams by slightly pulling out, and then up.
8. Check extraction well to ensure that no agarose is present, which could affect DNA yield and concentration.

9. Fill each buffer chamber with 5 ml of 0.25x TBE Buffer, except for the chamber to the left of the extraction well, which will be filled with 4 ml of buffer.
10. Place lid on cartridge.
11. Pipette 60 µL of 0.25x TBE buffer into the extraction well, and immediately seal with PCR tape, using the extraction well sealing tool to ensure a strong seal.
12. Turn on Thermocube (next to the Aurora) and press “start”.
13. The Thermocube can take a while to set. Make sure the “*” on the cube turns into a “+” or “-” when the start button is pushed. The asterisk means the thermocube temperature control is not active.
14. Don’t forget to check the level of the water in the Thermocube’s tank. Add more water if it needs.
15. Open the cartridge drawer on the Aurora instrument.
16. Place approximately 1 ml of Milli-Q water on the cold plate of the Aurora to ensure good thermal contact between the cartridge and cold plate.
17. Place the cartridge onto the cold plate oriented so the concentration gel is on the left hand side (see “Aurora Reusable Cartridge Handling Manual”).
18. Load the sample that passed the required amount of conductivity (100 µS/cm) into the ≤ sample chamber in the cartridge.

Tip:

*******As an added precaution to reduce the risk of DNA contamination, cut a piece of PCR tape large enough to cover the sample chamber.
Remove the white backing from the adhesive film and carefully lay it over the sample chamber from left to right, while leaving a small air gap between the concentration gel and the sample chamber to relieve any pressure during the run. Use a scalpel blade and forceps to cut away and remove any tape that may be covering any of the electrodes.
To seal the tape, use a PCR paddle or a flat object to seal the edges, take care to avoid any wrinkles or bubbles.***

19. Close the drawer and select the desired protocol.
20. Select the “106-0001-CA-D_AURORA_DNA_CLEAN-UP_PROTOCOL”. This protocol recovers DNA molecules 0.3-50 kb in length.
21. Create an experiment folder for the run if you want to save the logs.
22. The run will complete in 4 hours based on the selected protocol.

23. If you run the Aurora over night or the time that you won't be around after the run to obtain your cleaned sample, you can choose the same protocol with "wait-time" option. This option allows you to determine the time that you will be available for obtaining your sample. It basically postpones the focus process (the last step that concentrates and leads the DNA into the extraction well).
24. The initial conductivity check done by the Aurora may also takes a while, in which errors can occur. It is ideal to wait around for a short amount of time to make sure the run continues properly.

Tip:

*******When run is complete, extract purified DNA as soon as possible. DNA will begin to be reabsorbed by the gel if left too long.
If you're late to extract the sample, a block of refocusing can be done to try and recover any DNA loss that may have occurred.
Normal focus block is done for about an hour and a half, but the Focus Block protocol can be adjusted to desired length. The longer the focus, the (theoretically) higher DNA yield.***

25. When run is complete, open the drawer.
26. Examine the concentration gel and ensure that there are no large bubbles present. Presence of large bubbles indicates that the gel overheated during the run, which will adversely affect the quality of the output.
27. Using forceps to remove the PCR tape covering the extraction well.
28. Transfer concentrated DNA from this well using a micropipette.
29. Typically, two pipetting steps are required in order to extract most of the liquid from the extraction well. Be sure to extract any sample that is suspended on the walls of the extraction well. **The expected output volume is 50-60 µl, but may vary from 40-70 µl depending on sample and run conditions.**
30. Decant and dispose of the buffer from the cartridge. Using the cleaning brush, gently break up and remove the gel from the cartridge and rinse it with Milli-Q water.

Tip:

********It is very important to take care **not to scratch the bottom layer of plastic** in the cartridge.
Do not use brushes or other cleaning implements with sharp edges or points.

The layer of plastic separating the gel and buffer in a cartridge from the aluminum plate on the bottom of the cartridge is very thin, and if it is scratched it may be necessary to replace the cartridge.****

Please see the Aurora User Manual and Aurora Reusable Cartridge Handling Manual for troubleshooting information

******Optional Pre-Run***:***

This protocol is for removing any possible contaminating DNA in the Aurora cartridges. It is recommended for any sample that is expected to have low levels of DNA, and is run **prior to loading your sample and running a subsequent Aurora protocol of your choice**. Since the pre-run concentrates any contaminating DNA molecules within the cartridge, it ensures that contaminating DNA is not co-purified with the DNA from the sample of interest in the subsequent Aurora protocol.

1. Prepare the Aurora reusable cartridge like before, cast the gel, and fill the buffer chambers and extraction chamber with the same amount of 0.25x TBE buffer.
2. Don’t forget to seal the extraction well with with the PCR tape.
3. Load the sample chamber with 5 mL of ultra-pure water.
4. Place the cartridge in the Aurora drawer and select the “106-0017-AA-D Aurora Pre-Run Protocol.SP” file.
5. The run will complete in 1 hour and 30 minutes.
6. When the run is complete, carefully peel off the tape on the extraction well and remove the buffer.
7. Rinse the extraction well with 100 µL fresh buffer and discard.
8. Refill the extraction well with 60 µL fresh buffer and reseal the well with a new piece of tape.
9. Remove the water from the sample chamber, and refill the chamber with the intended sample.
10. Continue by selecting the protocol most appropriate for the sample.

Tip:

*******It doesn’t need to change the buffer in other chambers or recast the gel after this run.***

******Multiple Run***:***

1. Prepare the cartridge as normal, with fresh buffer in the extraction well.

2. Begin the run.
3. Pause the run after the Injection block as completed, near the beginning of the Wash Block.
4. Remove the buffer from the extraction well, replacing it with the extracted sample from previous run (bring the volume up to 60 µL with buffer prior to adding it to the extraction well).
5. Seal the extraction well with a fresh piece of PCR tape and resume the run.
6. Recover your sample at the end of the run, as usual.

Tip:

*******It is important that you **do not** begin the run with your previous run extracted sample in the extraction well, because during the Injection block that DNA will run off the back side of the gel into the buffer chambers and be lost.***

3. DNA Extraction from Sterivex Filters

Modified 2015 by the Brazelton Lab from protocols by Rika Anderson, Colleen Kellogg, Julie Huber, and Byron Crump. Incorporated some recommendations from Lever et al. (2015) Frontiers in Microbiology doi: 10.3389/fmicb.2015.00476. Contributions from Emily Dart and Lizethe Pendleton.

Do ahead of time:

Heat water bath or oven to 65ºC

Prepare solutions:

3 M sodium acetate, pH 5.2

DNA Extraction Buffer (DEB):	for 45 mL:
0.1M Tris-HCl (pH 8)	4.5 mL of 1.0 M
0.1M Na-EDTA (pH 8)	9 mL of 0.5M
0.1M KH_2PO_4 (pH 8)	0.54 g
1.5M NaCl	13.5 ml of 5M
0.8M Guanidine HCl	3.44 g
0.5% Triton-X 100	0.225 mL (225 µL) of 100%

Add above ingredients to 50 mL tube.

Add Milli-Q water to ~40 mL

Add NaOH to pH 10 (several drops at a time)
Add Milli-Q water to 45 mL
Filter-sterilize to remove possible spores
Autoclave. Slightly loosen lid so that it is not air-tight. Recover from autoclave very soon after the autoclave cycle is completed.
Pour autoclaved solution into fresh 50 mL tube.
Aliquot into 1.5 mL tubes.

Hot Lysis:

1. Add 1.4 mL of DEB to each Sterivex with syringe and needle. Position the needle just below the mouth of the Sterivex so that it does not come back out the top. Do not fill to the top – stop when solution covers white filter.

Possible Stopping Point. Store at -20ºC

2. Place sterivex filter in 50mL tube with holes.
3. Incubate at 65ºC for 30 min on Genemate spinning machine.
4. Vortex each sterivex again (inside the Falcon tube) for 30 seconds.

Bead Beating:

5. Using a syringe, withdraw fluid from each Sterivex and eject into bead tube (glass 0.1 mm for bacteria).
6. Bead beat for 40 s.
7. Centrifuge for 2 min at 5000 g.
8. Transfer fluid - avoiding beads - into fresh Eppendorf tube. Add no more than 900 µL in each tube (or no more than 750 µL if using 1.5 mL tubes).

Phenol / chloroform extraction:

9. Add equal volume of phenol / chloroform / isoamyl alcohol (25:24:1, bought pre-mixed with alkaline buffer) to each tube.
10. Gently shake a few times and then centrifuge at 14,000 x g for 1 minute.
11. Remove supernatant to fresh tube.
12. Add equal volume of chloroform / isoamyl alcohol (24:1) to each tube.
13. Gently shake a few times and centrifuge.
14. Remove supernatant to fresh tube, carefully avoiding the bottom organic layer.

Ethanol precipitation:

15. Redistribute aqueous phase among 3 tubes so that each 2.0 mL tube has 550 µL or less and each 1.6 mL tube has 450 µL or less. *For some samples, additional salt is not necessary, and you can skip the sodium acetate. In this case, you can add up to 600 µL in a 2.0 mL tube.*
16. Add 0.1 volumes sodium acetate (3M, pH 5.2). (*e.g*. add 55 µL to 550 µL.)
17. Add 2 volumes 100% ethanol. (*e.g.* add 1210 µL to 605 µL.)
18. [optional for low biomass samples] Add 1.2 µL of glycogen (20 µg/ µL).
19. Invert a few times to mix.
20. **Incubate at -20ºC for at least 1 hr. or overnight.** Incubation on ice might work just as well and yield a cleaner pellet.
21. Centrifuge for 40 minutes at 16,000 x g. (Optional: used cooled centrifuge at 0ºC)
22. Pour out supernatant. Do not completely invert tube; keep at a gentle angle to minimize the chance of the pellet falling out.
23. Add 500 µL of cold 70% ethanol to each tube.
24. Invert the tube to mix. Make sure the pellet is dislodged from the bottom so that it is properly washed.
25. Centrifuge at 16,000 x g for 10 minutes.
26. Remove liquid again with pipettor. Be careful to avoid pellet.
27. Place tubes with open lids in the Vacufuge. Spin for 7 minutes at 30ºC on the V-AL setting. If you can see ethanol in the tube, spin for another 2-5 minutes. If the pellets become powdery, they are too dry.
28. Resuspend in 100 µL of low EDTA TE. Heat to 55ºC for 10 or more minutes to dissolve pellet and store at 4ºC. For long-term storage, place at -20 or -80ºC, but avoid repeated freezing and thawing of the DNA. One strategy is to keep half at 4ºC for the working sample and store the other half at -80ºC as the archive sample.

Recipe for low EDTA TE:
10 mM Tris-HCl
0.1 mM EDTA

For 50 ml:
500 µl 1 M Tris-HCl (pH 8.0) autoclaved
10 µl 0.5 M EDTA (pH 8.0) autoclaved
→ to 50 ml with Milli-Q H_2O
→ filter sterilize with 0.22 µm syringe filter

TE is good for DNA storage, but EDTA inhibits PCR. So this low EDTA TE buffer is a good compromise for storing DNA for later PCR amplification. You can also just use EB (10 mM TrisHCl, pH 8 or 8.5).

4. Processing 16S rRNA gene amplicons with DADA2

Primary author was Christopher Thornton, with contributions from Shahrzad Motamedi, Cody Dangerfield, and Julia McGonigle

Note: The DADA2 tutorial and manual are very helpful references and sources for the workflow below.

Contents

- References
- Data Preparation
- Preprocessing
- Analysis
- Data Transformation

References

Callahan JB, McMurdie PJ, Rosen MJ, Han AW, Johnson AJ, Holmes SP. 2016. DADA2: High resolution sample inference from amplicon data. *Nature Methods*. doi: 10.1038/nmeth.3869.

Cole JR, Wang Q, Fish JA, Chai B, McGarrell DA, Sun Y, Brown CT, Porras-Alfaro A, Kuske CR, Tiedje JM. 2014. Ribosomal Database Project: data and tools for high throughput rRNA analysis. *Nucleic Acids Res.* 42(D1):D633-D642. doi: 10.1093/nar/gkt1244.

DeSantis TZ, Hugenholtz P, Larsen N, Rojas M, Brodie EL, Keller K, Huber T, Dalevi D, Hu P, Andersen GL. 2006. Greengenes, a Chimera-Checked 16S rRNA Gene Database and Workbench Compatible with ARB. *Appl. Environ. Microbiol.* 72:5069-72.

Martin M. 2011. Cutadapt removes adapter sequences from high-throughput sequencing reads. *EMBnet.journal* 17(1): 10-12. doi: 10.14806/ej.17.1.200.

McMurdie PJ, Holmes S. 2013. phyloseq: an R package for reproducible interactive analysis and graphics of microbiome census data. *PloS One* 8(4), e61217. doi:10.1371/journal.pone.0061217.

Wang Q, Garrity GM, Tiedje JM, Cole JR. 2007. Naive Bayesian classifier for rapid assignment of rRNA sequences into the new bacterial taxonomy. *Appl. Environ. Microbiol.* 73: 5261–5267. doi: 10.1128/AEM.00062-07.

Data Preparation

Starting Materials: Raw Illumina paired-end reads with partial to complete overlap in FASTQ format

Make a new project directory and create links to the appropriate raw data files:

```
mkdir -p <project>/short_reads
cd <project>/short_reads ln -svi
/path/to/fastq_files/* ./
```

Generate basic read statistics for each sample and remove any failed libraries:

```
srun readstats.py --csv -o readstats.csv ./* & less
readstats.csv mkdir failed_libs mv
./<files_with_very_low_counts> failed_libs/
```

Create a workspace to process the data in:

```
mkdir  ../preprocess
cd ../preprocess
```

Discard contaminant sequences:

a. Create a slurm batch file named decontam.sh with the following content (replace **<text>** with the appropriate information):

```
#! /bin/sh
#SBATCH -J <project>.decontam
reads_path="</path/to/short_reads>";
files=$(find -L ${reads_path} -type f -regex ".*\.20.forward\.fastq.*");
for file in ${files[@]}; do
```

```
    file=$(basename ${file});
    IFS=". " read -a array <<< ${file};
    sample=${array[0]};
    forward="${reads_path}/${sample}.20.forward.fastq.gz";
    reverse="${reads_path}/${sample}.20.reverse.fastq.gz";
    cutadapt --discard-trimmed --error-rate 0.10 -a ATTAGAWACCCVHGTAGTCCGGCTGACTGACT -A
TTACCGCGGCMGCTGGCACACAATTACCATA -g ^TTAGAWACCCVHGTAGTCCGGCTGACTGACT -G
^TACCGCGGCMGCTGGCACACAATTACCATA -o ${sample}.20.forward.decontam.fastq.gz -p $
{sample}.20.reverse.decontam.fastq.gz ${forward} ${reverse} > ${sample}.cutadapt.log
done
```

b. Execute the batch script

```
sbatch decontam.sh
```

* in amplicon sequence data, contaminants tend to come from the primer sequences used to amplify a region of the genome.

* assumes primers 515f and 806r as described in Kozich et al. 2013 were used to amplify the V4 region ofthe 16S rRNA gene. If not, the appropriate sequences will need to be provided to cutadapt arguments -a/A and -g/-G. Use fastqc to determine potential adapter and primer sequences that need to be removed. See srun cutadapt --help for details on usage. Note that forward primers tend to be found in reverse reads and reverse primers in forward reads.

Confirm that most of the reads in each sample passed the sequence contamination filters:

```
grep "Pairs written (passing filters):" *.log > passed_filter.txt
less passed_filter.txt
```

* if only a low percentage passed, you may consider removing the sample from the dataset.

Create a screen session for the project:

```
screen -S <project>
```

Start R and load the required libraries:

```
srun --x11=first --pty R
library('dada2')
library('ShortRead')
library('ggplot2')
library('grid') library('gridExtra')
```

Set the appropriate path variables:

```
path <- getwd()
reads <- list.files(path)
```

Select only the fastq files and sort them so that the forward and reverse reads are in the same order:

```
fastqs <- sort(reads[grepl('.fastq+', reads)])
ff <- fastqs[grepl('forward', fastqs)]
rf <- fastqs[grepl('reverse', fastqs)]
```

Obtain a list of sample names:

```
samples <- sapply(strsplit(ff, '[.]'), '[', 1)
```

* takes the nth item of a string after being split by a delimiter. Assumes that files are delimited by a period, with the sample name as the prefix (e.g. 0AMRd001.forward.fastq). If a character other than a period is used as the delimiter, insert the appropriate character into the second argument of strsplit.

Add full paths to the files:

```
ff <- paste0(path, '/', ff)
rf <- paste0(path, '/', rf)
```

Examine a sampling of the dataset's quality profile:

```
n <- sample(length(ff), <num_samples>)
forward_plots <- list()
reverse_plots <- list()
for (i in 1:length(n)) {
sample_index <-n[i]
  fp <- plotQualityProfile(ff[sample_index])
 rp <- plotQualityProfile(rf[sample_index])
 fp <- fp + ggtitle(samples[sample_index])
  rp <- rp + ggtitle(samples[sample_index])
  forward_plots[[i]] <- fp
 reverse_plots[[i]] <- rp
 }
 grid.arrange(grobs=forward_plots)
 grid.arrange(grobs=reverse_plots)
```

* the diagrams show the distribution of quality scores as a function of sequence position. Take note of where the drop in quality tends to occur, as this information will be used in later preprocessing steps. If it is not clear from using num_samples samples,

additional samples can be plotted to get a better idea of the quality trends of the dataset as a whole.

* the plotted lines are the summary statistics for each position: solid green reflects the mean, solid orange the median, and dashed orange the 25th and 75th quantiles.

Set additional path variables:

```
ff.filt <- paste0(path, '/', samples, '.forward.decontam.filtered.fastq.gz')
rf.filt <- paste0(path, '/', samples, '.reverse.decontam.filtered.fastq.gz')
```

Preprocessing

Note: Preprocessing should be done separately when samples are from different sequencing runs and then merged later, if desired.

Trim sequences and filter by quality:

```
filtered <- filterAndTrim(ff, ff.filt, rf, rf.filt, maxN=0, maxEE=3, truncQ=2, compress=TRUE, verbose=TRUE,
matchIDs=TRUE, rm.phix=c(TRUE, TRUE))
```

Combine identical sequences to retain only the uniques:

```
ff.derep <- derepFastq(ff.filt, verbose=TRUE)
rf.derep <- derepFastq(rf.filt, verbose=TRUE)
```

Add sample names to the derep-class objects:

```
names(ff.derep) <- samples names(rf.derep) <-
samples
```

Estimate error rates to be used in the dada algorithm:

```
ff.err <- learnErrors(ff.derep[n], errorEstimationFunction=loessErrfun, randomize=FALSE)
rf.err <- learnErrors(rf.derep[n], errorEstimationFunction=loessErrfun, randomize=FALSE)
```

* n, the same sample subset that was generated above, should be large enough to adequately represent the dataset as a whole. It is recommended to start with a small fraction of the total sample size (~10% for large datasets, 20-30% for small) and then use the error plots generated in the next step to determine if the rates have been sufficiently estimated. If not, generate a new subset with n <- sample(length(ff), **<num_samples>**) and rerun the command.

Visualize the estimated error rates:

```
plotErrors(ff.err, nominalQ=TRUE) plotErrors(rf.err,
nominalQ=TRUE)
```

* plots the observed frequency of each transition and transversion (observed error rates) as a function of quality score. The red line represents the expected error rates. The black line represents the fitted rates after convergence.

* the plots should be used to verify that the error rates have been sufficiently estimated. Visually, this can be determined by how well the model (black line) tracks the observed error rates (the black dots). Note that this does not mean that the line needs to pass through all the dots; often a more general model is a better predictor than one that fits the data extremely well. If the error model does not provide a sufficient fit, re-run the parameter estimation step with a larger sample subset.

Infer sequence variants from the dataset:

```
ff.dada <- dada(ff.derep, err=ff.err, pool=TRUE) rf.dada <-
dada(rf.derep, err=rf.err, pool=TRUE)
```

* samples should be pooled (pool=TRUE) whenever possible as it allows information to be shared across samples, making it easier to resolve rare variants. Samples should not be pooled, however, unless they share a similar error 'history'. Cases in which samples will not have a shared error history include when they come from different sequencing runs or if different PCR protocols were used for amplification. In these cases, dada should be run with the parameter selfConsist=TRUE or separately for the different sample sets. The memory requirements are also larger for pooled samples, so it may be necessary to run the command with selfConsist=TRUE for very large datasets (>200 samples).

Inspect the dada-class objects:

```
ff.dada rf.dada
```

* see help('dada-class') for the kinds of information accessible from dada-class objects.

Merge overlapping paired-end reads:

```
merged <- mergePairs(ff.dada, ff.derep, rf.dada, rf.derep, verbose=TRUE,
maxMismatch=0, trimOverhang=FALSE, justConcatenate=FALSE)
head(merged[[1]])
head(merged[[2]])
```

Construct a sequence-by-sample table:

```
seqtable <- makeSequenceTable(merged, orderBy='abundance')
dim(seqtable) table(nchar(colnames(seqtable)))
```

Remove chimeric sequences:

```
seqtable.nochim <- removeBimeraDenovo(seqtable, method="consensus", verbose=TRUE)
dim(seqtable.nochim)
```

Determine how many total sequences are retained after each step:

```
getN <- function(x) sum(getUniques(x))
track <- cbind(filtered, sapply(ff.dada, getN), sapply(merged, getN), rowSums(seqtable.nochim))
colnames(track) <- c("raw", "q-filtered", "denoised", "merged", "chimera-checked")
rownames(track) <- samples
track
```

Analysis

Create a workspace to analyse the data:

```
path <- '/path/to/project_dir/analysis/' system(paste('mkdir', path))
```

Assign taxonomy to the inferred, chimera-filtered sequences:

```
refdb <- /srv/databases/markers/silva/dada2/silva_nr_v132_train_set.fa.gz
taxa <- assignTaxonomy(seqtable.nochim, refdb, tryRC=FALSE, minBoot=50, verbose=TRUE)
colnames(taxa) <- c("Domain", "Phylum", "Class", "Order", "Family", "Genus")
```

Merge the different components of the data into a phyloseq-class object:

```
library('phyloseq')
meta.table <- read.table("<meta-data table>", sep=',', header=TRUE, row.names=1)
<project>.dat <- phyloseq(otu_table(seqtable.nochim, taxa_are_rows=FALSE), tax_table(taxa),
sample_data(meta.table))
```

Output processed data to files:

```
fasta.out <- paste0(path, '/<project>.preprocessed.fasta')
seqtable.out <- paste0(path, '/<project>.seqtable.tsv')
taxa.out <- paste0(path, '/<project>.taxonomy.tsv')
unqs <- getUniques(seqtable.nochim)
uniquesToFasta(unqs, fasta.out)
ids <- paste0("sq", seq(1, length(unqs)), ";size=", unname(unqs), ";")
seqtable.tmp <- t(seqtable.nochim)
 row.names(seqtable.tmp) <- ids
write.table(seqtable.tmp, file=seqtable.out, quote=FALSE, sep="\t", row.names=TRUE,
col.names=TRUE) taxa.tmp <-
taxa row.names(taxa.tmp) <-
ids
```

```
write.table(taxa.tmp, file=taxa.out, quote=FALSE, sep="\t", row.names=TRUE, col.names=TRUE)
```

Merge the taxonomy and otu table into one file for convenient browsing:

```
srun count_cat_tax_csv.py -t tax-table.csv -c otu-table.csv -o otu-tax-table.csv
```
